# Gastral Drumming in *Vespula germanica* (Hymenoptera: Vespidae): Vibrational Communication at Night Suggests Additional Roles in Colony Organization

**DOI:** 10.1093/jisesa/ieac030

**Published:** 2022-05-13

**Authors:** Benjamin J Taylor

**Affiliations:** Department of Natural Sciences, City University of New York-LaGuardia Community College, Long Island City, NY 11101, USA

**Keywords:** communication, substrate vibration, recruitment, modulatory signals, social cues

## Abstract

Gastral drumming (GD) is a type of vibrational communication that has been reported in several species of yellowjackets and hornets. Despite early claims that it acts as a hunger signal, a more recent study found evidence that it acts as a nest-based food-recruitment signal, the first reported for eusocial wasps. Early studies also claimed, without supporting data, that it is produced most often in the early morning hours when the sun rises. Here, I recorded drumming continuously in colonies of *Vespula germanica* (Fabricius) to assess whether production was highest in the morning. Although I found no evidence in support of greater early morning production, I found, surprisingly, that it is produced at night, a time when foraging does not occur. When these results are combined with the results from previous studies on this species and similar findings in honey bees, they suggest that GD may be a modulatory signal, which acts by increasing general activity levels and by increasing the rate that individuals come into contact with social cues.

Social insects are highly successful and much of their success can be attributed to their ability to coordinate large numbers of individuals to carry out group tasks (Oster and Wilson 1978, Wilson 1990). Central to the ability of a colony to coordinate its activities is the flow of information among its members via signals and cues. Some signals are produced in a specific context and elicit a particular behavioral response in the receiver. Modulatory signals, in contrast, act by altering the motivational state of the receiver, causing it to adjust its behavior towards what is appropriate in accordance with surrounding environmental cues or other signals (Hölldobler 1999).

Social insects coordinate group tasks using a variety of modes of communication. Although the chemical mode is considered the primary means by which social insects communicate (Richard and Hunt 2013), vibroacoustic signals are also used (Hunt and Richard 2013, Taylor and Jandt 2020). Gastral drumming (GD) is one such vibroacoustic signal that is found in vespine wasps (Ishay et al. 1974, Ishay 1975, Taylor and Jeanne 2018). Taylor and Jeanne (2018) described this behavior in detail for *Vespula germanica* (Fabricius). A drum consists of a series of 8–28 strikes of the gaster against the nest. Drums are separated by short (~2 s) inter-drum intervals, and each series of drums by a single individual is termed a bout. Ishay and colleagues hypothesized that GD communicates hunger because it was mostly produced in the morning after a night without food, claiming that the highest levels occurred in the early morning when the sun rises (Ishay 1975, Ishay and Nachsen 1975, Ishay and Sadeh 1982, Barenholz-Paniry and Ishay 1988). Shining a flashlight into the nest was said to produce the same effect (Ishay and Schwartz 1973). Further, GD production was said to increase when workers were starved by preventing them from exiting the nest (Ishay and Sadeh 1982). Unfortunately, these studies did not provide any supporting data. In contrast, Taylor and Jeanne (2018) found that drumming rates increased when colonies were supplemented with carbohydrate food and decreased when colonies were starved. In addition, GD playback resulted in increased foraging departures, worker movement, and worker-worker trophallaxis. Collectively, these results suggest that GD acts as a recruitment signal to food resources and is not a hunger signal (Taylor and Jeanne 2018).

In the present study, drumming from *V. germanica* colonies was recorded continuously over 24-h periods to determine whether spikes in GD production actually occurred in the early morning, as suggested in previous studies (Ishay 1975, Ishay and Nachsen 1975, Ishay and Sadeh 1982, Barenholz-Paniry and Ishay 1988).

## Materials and Methods

### Nest Excavation and Setup

Two queenright colonies were anesthetized with anhydrous ether and excavated in Dane County, Wisconsin, USA. Colony 1 was excavated in Madison, Wisconsin on 3 August 2010, and Colony 2 was excavated in Lodi, Wisconsin, on 25 August 2010. Each colony contained only a queen and workers at the time of excavation. The nest envelopes were removed, and the queen and workers were collected and placed in a refrigerator overnight. Combs were separated and glued via wooden dowel to the ceilings of a nest box built to the specification of Taylor et al. (2012), except that the Masonite ceiling was replaced with Plexiglas. The dowel spacers provided room for the workers and queen to crawl between the comb and Plexiglas. A data logger (Lascar Electronics EasyLog USB-2, Whiteparish, Wiltshire, UK) was used to record temperature and relative humidity every hour inside each nest box. The day after collection, the queen and workers were placed into the nest box and sealed inside by sliding the Plexiglas bottom into place. After the colonies roused from torpor, they acted similar to a colony responding to a disturbance. However, this subsided within a couple of hours, and by the next day activities such as tending brood, comb and envelope construction, and foraging for food resumed. The colonies did not appear stressed during the time they were observed and both colonies continued to produce new brood, including new gynes near the end of the season.

Colonies were kept on the fifth floor of the Russell Laboratories building in Madison, Wisconsin (43.0755°N, 89.413°W). Workers had access to outside foraging via a Tygon (Saint-Gobain S.A., Courbevoie, France) tube (2.5 cm inside diameter), which led from the nest box through a plywood panel that covered an open window. The nest box and tube were double wrapped in thick black velvet throughout the experiment to block ambient light.

### Vibration Recordings

To detect GD, an accelerometer (Brüel and Kjær type 4366) was hot glued to the Plexiglas top of the nest box, centered over the combs. The signal from the accelerometer was amplified (Brüel and Kjær charge type 2635), and the output was recorded on a computer using Audacity. Sensitivity of the accelerometer was set at 4.26 pC/(m/s^2^), and the output was amplified 100x to measure 0.01 (mm/s^2^)/mV. Before recordings began, I confirmed that the accelerometer was picking up drumming in all parts of the nest.

In all, 144 h of activity were recorded (Colony 1: 72 continuous hours from 1100 h on 28 August 2010 until 1100 h on 31 August 2010; Colony 2: 72 continuous hours from 2300 h on 9 September 2010 until 2300 h on 12 September 2010). During each day of recording, sunrise occurred during the hour of 0600–0700 h, and sunset during the hour of 1900–2000 h. The recordings were saved as. wav files for later analysis.

### Data Collected and Statistical Analyses

The audio files were analyzed using Audacity. Initially, I planned to record both the number of worker bouts produced each hour and total time spent drumming per hour. However, I found that GD bouts by single individuals could not always be reliably identified because bouts from multiple individuals sometimes overlapped. Consequently, I used Audacity to highlight the time during each hour that one or more drums were in progress. Totaling the highlighted times for each hour yielded a colony-wide measure of minutes of drum per hour by one or more individuals.

A video recording was made of the top and bottom of each nest to get an estimate of colony populations. Colony 1 was recorded on 26 August 2010 (2 d before audio recordings began). Colony 2 was recorded on 14 September 2010 (2 d after audio recordings ended).

PROC MIXED in SAS version 9.4 for Windows (SAS Institute Inc., Cary, NC) was used to analyze the results under a mixed-effects model. The model had five factors. Of these, hour (of day) was treated as a fixed, categorical factor, whereas both temperature and relative humidity were treated as fixed, continuous factors. Colony and date were modeled as random factors. In addition, a contrast tested whether the hour associated with sunrise differed from the rest of the day, as suggested by previous studies.

## Results

**Fig. 1.**
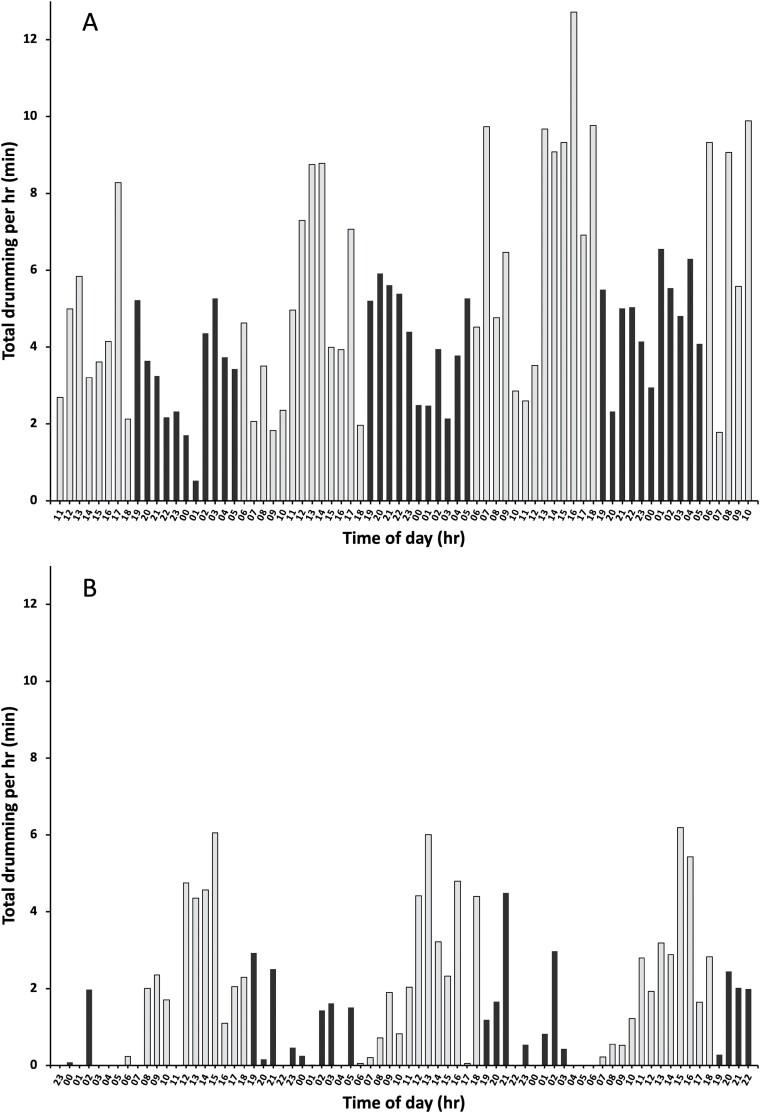
Daily drumming pattern, shown as the total time of drums (both individually and group-produced) within each hour from 8/28 to 8/31 in 2010 for (A) Colony 1 and from 9/9 to 9/12 in 2010 for (B) Colony 2. Both colonies were recorded for a total of 72 h. The two numbers below each bar on the x-axis represent the starting time for each hour (e.g., 11 represents the hour 1100–1200). The grey bars represent daylight hours, whereas the black bars represent nighttime. Note that for (A) Colony 1 and (B) Colony 2, the x-axis starts and ends at different times due to the different start times of the recordings.

In all, 32,171 drums were recorded, 24,391 from Colony 1 and 7780 from Colony 2. The drums ranged in length from 0.03 s to a large, group-produced overlapping drum that lasted 7.41 s. Drumming activity was variable during the 24-h cycle and differed between the colonies. Colony 1 produced at least some drumming during all hours of the day (Fig. 1A), whereas the smaller Colony 2 had several hours where no drumming occurred (Fig. 1B). From the video scans of the nest, the population of Colony 1 was estimated at 514 workers, whereas Colony 2 had 353 workers.

**Fig. 2.**
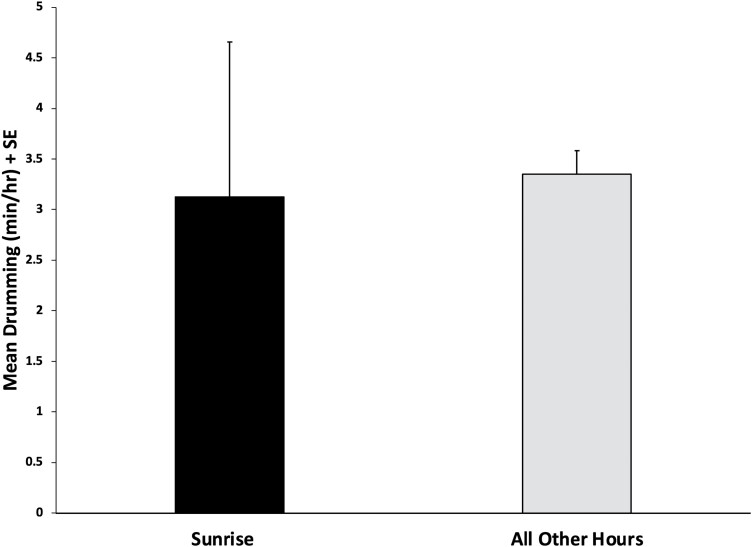
Results from the contrast comparing GD production during the hour of sunrise vs. all other hours of the day. Error bars represent standard error of the mean. There was no statistically significant difference (*P* = 0.240).

**Fig. 3.**
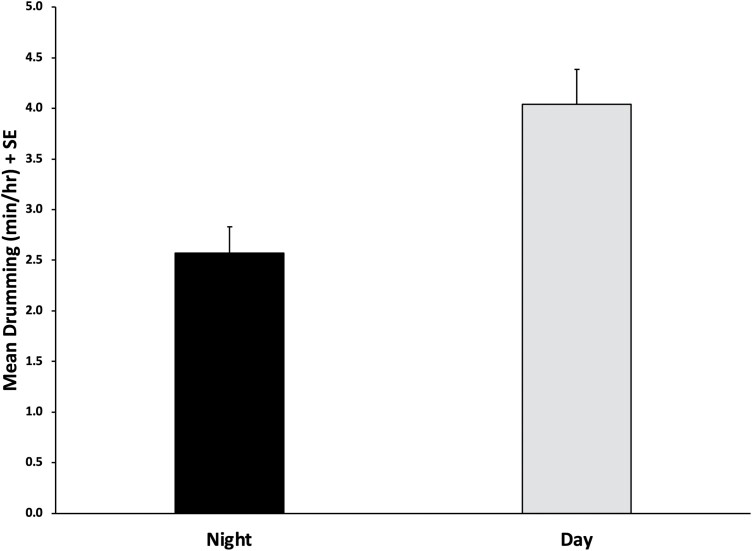
Results from the contrast comparing GD production during the day vs. production at night. Error bars represent standard error of the mean. The contrast revealed significantly higher production during the day (*P* < 0.001).

From the mixed-effects model, significant effects of hour (*F*_23,111_ = 2.09, *P* = 0.006) were found, but there were no effects of either temperature (*F*_1,111_ = 0.00, *P* = 0.966) or relative humidity (*F*_1,111_ = 0.87, *P* = 0.352). The contrast indicated that there were no differences in drumming rates between the hour of sunrise and all other hours (*F*_1,111_ = 1.40, *P* = 0.240, Fig. 2). Because drumming was recorded at night for both colonies, an additional contrast was run to determine if it occurred more often during the day or the night. The results from the contrast indicated that daytime levels were higher than those at night (*F*_1,111_ = 18.66, *P* < 0.001, Fig. 3).

## Discussion

In contrast to prior studies on this species (Ishay 1975, Ishay and Nachsen 1975, Ishay and Sadeh 1982, Barenholz-Paniry and Ishay 1988), I did not find that the highest levels of drumming production occurred in the early morning. It is unclear what might have led to differences in what was observed here vs. those prior studies, as the earlier studies provided no data describing the production throughout the day. The most significant finding presented here is that GD also occurs at night (albeit at lower than daytime rates), a time when no foraging occurs. The results from a previous study suggested that GD acts as a food recruitment signal (Taylor and Jeanne 2018). The results shown here suggest that the function of GD is more complicated than previously thought and indicate that it is produced in contexts other than incoming food.

One clue to the function of this behavior may be provided by studies of the vibration signal in honey bees. The honey bee vibration signal [also known as dorso-ventral abdominal vibration (DVAV) or the shaking signal] also occurs during the night (Schneider and Lewis 2004), and may in fact occur more frequently at night (Ramsey et al. 2018). It is sometimes produced in the pre-dawn hours, especially if the colony has experienced several days of profitable foraging (Allen 1959, Schneider et al. 1986a,b, Nieh 1998, Seeley et al. 1998). This signal is also produced in a variety of other contexts, including queen-replacement, swarm emigration (particularly during the hour prior to swarm lift-off), and when a profitable foraging opportunity presents itself following a food dearth (Schneider 1991, Schneider et al. 1998, Seeley et al. 1998, Visscher et al. 1999, Lewis and Schneider 2000). Its effects are varied and depend on the context in which the signal is produced and on the age of the receivers (Schneider and Lewis 2004). However, vibrated recipients tend to increase their rates of movement, trophallaxis, and cell-inspection. They also often move to the dance floor, where they are likely to encounter waggle dancers and foraging-related cues, which leads to an increased probability of foraging (Schneider and Lewis 2004, Cao et al. 2007, Hyland et al. 2007, Koenig et al. 2020). During emigration, vibrated individuals show an increased probability of flying from the swarm to look for nest sites or depart for a nest site (Schneider et al. 1998, Visscher et al. 1999, Lewis and Schneider 2000, Donahoe et al. 2003). Overall, the vibration signal seems to have a modulatory effect on recipients, preparing them for greater activity and increasing the rate at which they come into contact with social cues, which in turn improves task efficiency (Seeley et al. 1998, Schneider and Lewis 2004, Cao et al. 2007, Hyland et al. 2007). I suggest that GD in *Vespula* and the vibration signal in *Apis* may serve similar functions, i.e., acting as a modulatory signal and increasing the rate at which social cues are encountered. In line with this hypothesis, GD has been found to increase both rate-of-movement and trophallaxis in workers, which in turn would bring them into more frequent contact with social cues (Taylor and Jeanne 2018). Further, Taylor and Jeanne (2018) found that rates of departures from the nest increased when the social cue of food was presented to the nest, compared to the signal of GD alone, suggesting that food-associated social cues are important for increasing foraging rates. It is possible that rates of other in-nest activities increase when GD is produced during the night, and that the activities are dependent on the types of social cues that workers encounter. Indeed, Ishay et al. (1974) reported that GD playback resulted in a general intensification of activities within the nests of *Vespa orientalis* (Fabricius), including worker visits to larvae and workers departing on foraging trips, but did not provide any data. GD’s effects on work rates other than foraging remain unknown.

It is worth noting that stress in honey bee colonies also results in increased foraging. Honey bee colonies exposed to a variety of stressors induces precocious foraging in workers, which may lead to decline of the colony (Klein et al. 2017). Is GD also a stress signal? Most of the evidence suggests that it is not. No manifestations of stress, such as cannibalization of brood, were observed in the colonies during the days immediately prior to or after they were observed in this study. As far as is known, both colonies behaved normally, and foraging for food, tending brood, oviposition by the queen, and nest construction all resumed within one day of transplanting the colony into the nest box. Furthermore, both colonies produced new gynes. However, a stronger test of the stress signal hypothesis could be accomplished by exposing colonies to various stressors to see if drumming rates increase.

Interestingly, the dorso-ventral movements in honey bees and yellowjackets are similar, but the methods of transmission differ. Although a honey bee grasps an individual worker and shakes her, yellowjackets strike the nest itself. These differences may arise from adaptations to the different wave transmission properties of each nest material. Although the wax comb of honey bees transmits vibrations, these attenuate rapidly from the point of incidence, especially in framed hives containing brood (Ishay 1975, Kirchner 1993, Sandeman et al. 1996). In contrast, the paper nests of wasps are particularly good at transmitting vibrations (Hunt and Richard 2013).

Other species of social wasps also produce vibrations on the nest. Aside from the modulatory signal mentioned above, the contexts in which they are produced fall into four categories. Scraping in *Chartergus*, *Synoeca*, *Asteloeca ujhelyii*, *Chartergellus golfitensis*, and gaster tapping in *Parachartergus colobopterus* and some species of *Polistes* appear to be alarm signals in response to disturbances (Hunt and Richard 2013, Taylor and Jandt 2020). This seems an unlikely function for GD in *V. germanica* since disturbances were avoided throughout this study, yet GD was produced consistently throughout the day. Buzzing/breaking runs are produced in swarm-founding epiponine wasps and may signal to the colony that it is time to move to the new nest site (Sonnentag and Jeanne 2009, Hunt and Richard 2013, Taylor and Jandt 2020). Again, GD would not fall into this category since *Vespula* wasps do not swarm. Lateral vibrations, antennal drumming, and abdominal wagging in *Polistes*, longitudinal vibrations in *Mischocyttarus drewseni*, *Ropalidia marginata*, and *Belonogaster petiola*, and gastral drumming in *Mischocyttarus drewseni* are all produced in the context of feeding larvae (reviewed in Hunt and Richard 2013, Taylor and Jandt 2020). As mentioned above, Ishay et al. (1974) indicated that GD playback resulted in a greater number of visits to larvae. However, this claim should be interpreted with caution since no supporting data were provided. Furthermore, at least three types of vibration thought to be associated with feeding larvae—antennal drumming, abdominal wagging, and lateral vibration—have been implicated in biasing larvae towards a worker-like role (Brillet et al. 1999, Jeanne 2009, Suryanarayanan and Jeanne 2011, Mignini and Lorenzi 2015). Caste determination in vespine wasps is thought to be the result of food amount, perhaps coupled with differences in pheromones or secretions fed to larvae (Greene 1991). However, in line with the caste-biasing hypothesis, it would be expected that drumming would not be restricted to only the daytime hours. Indeed, much like GD, antennal drumming in *Polistes* has been observed at night (Pratte and Jeanne 1984). Furthermore, the caste-biasing hypothesis predicts that drumming would decline towards the end of the season as gynes are produced. In support of this hypothesis, I found that Colony 2, recorded later in the season, produced less GD than Colony 1, which was recorded earlier. However, Colony 1 was also larger than Colony 2, which could account for the differences in drumming rates. In addition, both colonies produced new gynes and continued to drum, which does not support this hypothesis. A longer, longitudinal study examining drumming rates across the season could well resolve this question.

Another possible role for GD is that it draws workers to individuals that possess food resources. The individuals that are drawn to the food may then react differently depending on the context. During the day, these individuals may depart on foraging trips after obtaining food, whereas during the night they would simply feed. *Vespula* spp. do not store food in the nest (Jandt and Jeanne 2005) and live in dark nests underground. Thus, GD may provide a way for hungry workers at night to find nestmates with food in their crops or to gather/transmit information during the day. Additional studies are required to test this hypothesis. Regardless, the role this behavior may play in colony organization has clearly been understudied. Future observational studies are needed to elucidate the contexts in which it is produced, combined with manipulative experimental studies to determine its function.
